# An induction heating system for *in situ* X-ray diffraction imaging: design, simulation and application to dislocation dynamics in semiconductors

**DOI:** 10.1107/S1600577526004728

**Published:** 2026-06-05

**Authors:** Merve P. Kabukcuoglu, Nikolaos Sagias, Elias Hamann, Carsten Richter, Marcus Zuber, Kaspars Dadzis, Daniel Hänschke

**Affiliations:** a Leibniz-Institut für Kristallzüchtung (IKZ) Max-Born-Str. 2 12489Berlin Germany; b Institute for Photon Science and Synchrotron Radiation (IPS) Karlsruhe Institute of Technology (KIT) 76344 Eggenstein-Leopoldshafen Germany; c Institute of Electrotechnology (ETP) Leibniz University Hannover Wilhelm-Busch-Str. 4 30167Hannover Germany; European XFEL, Germany

**Keywords:** induction heating system, X-ray diffraction imaging, numerical simulations, dislocation dynamics

## Abstract

We present a compact induction heating system for time-resolved *in situ*X-ray diffraction imaging, enabling contact-free volumetric heating of samples up to approximately 1600°C with flexible operation in different working modes. Real-time, spatially resolved thermography is achieved using an integrated near-infrared camera. A three-dimensional finite-element model of electromagnetic heating, steady-state heat transfer and thermo-elastic stress predicts Joule heating, temperature fields and resolved shear stresses, and guides experimental design. The system has been demonstrated at a synchrotron topography station using simultaneous X-ray white-beam topography and infrared thermography during controlled heating of an indented Si(001) wafer. Dislocation activity is observed starting at local temperatures above 1000°C, increasing at higher temperatures. The experimentally observed number of dislocations on individual {111}〈110〉 glide systems correlates with the simulated resolved shear stresses. This integrated approach enables quantitative, time-resolved studies of dislocation dynamics under well defined thermal conditions and offers a simulation-guided route to tailoring temperature gradients and stress fields for future materials and *in situ* experiments.

## Abstract 2

This work presents a compact induction heating system for time-resolved *in situ* X-ray diffraction imaging. The system enables contact-free volumetric heating up to approximately 1600°C and provides flexible operation in different operating modes. Its performance is demonstrated through representative diffraction imaging experiments.

## 1. Introduction

Thermal treatments play a fundamental role in semiconductor processing; for example, they are used to activate dopants (Boyd & Wilson, 1980 ▸), remove native oxides (Yamazaki *et al.*, 1992 ▸) and relieve residual stresses (Scheiter *et al.*, 1996 ▸). Understanding how crystalline defects such as dislocations evolve during such treatments – particularly how they nucleate, propagate and interact under thermo-mechanical stresses – is essential for predicting the impact of industrial processes and, ultimately, for ensuring device reliability through improved process control (Yonenaga, 2005 ▸).

Conventional defect characterization, such as chemical etching, polarized light microscopy or metal-decoration techniques (Hull & Bacon, 2011 ▸), can reveal such dislocations but are limited to near-surface regions or require optically transparent samples. Electron microscopy provides two-dimensional (2D) and three-dimensional (3D) images of dislocations with nanoscale resolution, but is restricted to small volumes and requires destructive sample preparation (Hirsch *et al.*, 2006 ▸; Chen *et al.*, 2013 ▸). Consequently, their applicability to time-resolved *in situ* studies of bulk materials with industrially relevant dimensions remains limited.

These constraints are addressed by synchrotron-based X-ray diffraction imaging – a non-destructive, *in situ* capable method for micrometre-scale characterization of wafer-sized crystal volumes, building on early developments in X-ray topography for *in situ* defect observation demonstrated by Chikawa (1974 ▸). Among diffraction-imaging techniques, X-ray white-beam topography (XWBT) (Tuomi *et al.*, 1974 ▸) is particularly well suited for time-resolved *in situ* studies. Unlike monochromatic X-ray diffraction imaging, XWBT does not require high-precision alignment and is thus largely tolerant of heating-induced effects such as thermal expansion (with local changes of the Bragg condition) and thermal drift of the sample and setup. For example, *in situ* XWBT was performed during heating with halogen lamps, focused on the sample using a double-ellipsoidal mirror system (Danilewsky *et al.*, 2011*a* ▸), demonstrating its potential for studying dislocation nucleation and slip-band formation (Wittge *et al.*, 2010 ▸; Danilewsky *et al.*, 2011*b* ▸) and crack propagation (Danilewsky *et al.*, 2013 ▸) in silicon (Si) wafers at temperatures up to approximately 1000°C.

A variety of heating systems are employed for synchrotron *in situ*X-ray diffraction imaging experiments, including resistive heating (Fan *et al.*, 2010 ▸; Lesage *et al.*, 2026 ▸), laser heating (Andrault & Fiquet, 2001 ▸), radiative or optical furnaces (Danilewsky *et al.*, 2011*a* ▸; Tsoutsouva *et al.*, 2018 ▸; Yildirim *et al.*, 2020 ▸) and inductive heating (Kudrna Prašek *et al.*, 2018 ▸). These approaches differ in terms of their heating mechanisms, achievable temperature ranges, their compatibility with beamline geometries, and the specific requirements of the targeted materials and *in situ* experiments. Within this framework, the present study introduces a contact-free induction heating system that enables volumetric heating in an open geometry ideally suited for *in situ* synchrotron experiments, combined with real-time thermal imaging. The system provides fully programmable heating profiles, rapid temperature response (<1 s) and a wide operating range up to 1600°C. The integrated infrared (IR) camera facilitates the correlation of thermal data with dislocation dynamics from X-ray diffraction imaging during heating. While operable as a standalone mobile instrument, it also interfaces with typical synchrotron beamline infrastructure, enabling unified experimental control and simultaneous operation of thermal treatment, X-ray diffraction and thermal imaging to directly correlate dislocation dynamics with the evolving temperature field.

In order to investigate thermally activated dislocation dynamics under controlled conditions, we use a silicon wafer that contains a defined 10 × 10 array of Berkovich indentations, which act as well defined and localized sources of dislocations. This approach enables a direct correlation between spatio-temporal evolution of the temperature field with development of dislocations during *in situ*X-ray diffraction imaging, while an integrated near-IR camera provides spatially and temporally resolved thermal monitoring of the sample surface.

To interpret the experimentally observed dislocation dynamics in terms of the underlying driving forces, we employ a 3D finite-element model that calculates the time-harmonic electromagnetic field to account for Joule heating by induced eddy currents, solves steady-state heat conduction within the sample and its environment, and computes the resulting thermo-mechanical stresses. In particular, the simulations provide the spatial distribution of the resolved shear stress (RSS), which serves as a quantitative measure for dislocation glide and enables identification of active glide systems. This simulation framework allows the reproduction of the measured temperature field and supports the design of the complex experiments. Finally, we demonstrate the capabilities of the induction heating system through combined *in situ* and *ex situ* investigations of thermally driven dislocation dynamics in a nano-indented Si(001) wafer, correlating the observed dislocation activity on each glide system with the calculated RSS to quantify the resulting micro-structural evolution and the underlying thermo-mechanical driving forces.

## 2. Instrumentation: induction heating system

The induction heating system is designed for both standalone laboratory operation and integration into synchrotron beamlines, such as for X-ray diffraction imaging experiments. It operates in ambient air but can be adapted for operation under controlled atmospheres if required for experiments. The open geometry allows simultaneous X-ray diffraction imaging of the sample while the opposite surface is monitored by the infrared camera during synchrotron experiments.

**Figure 1:**
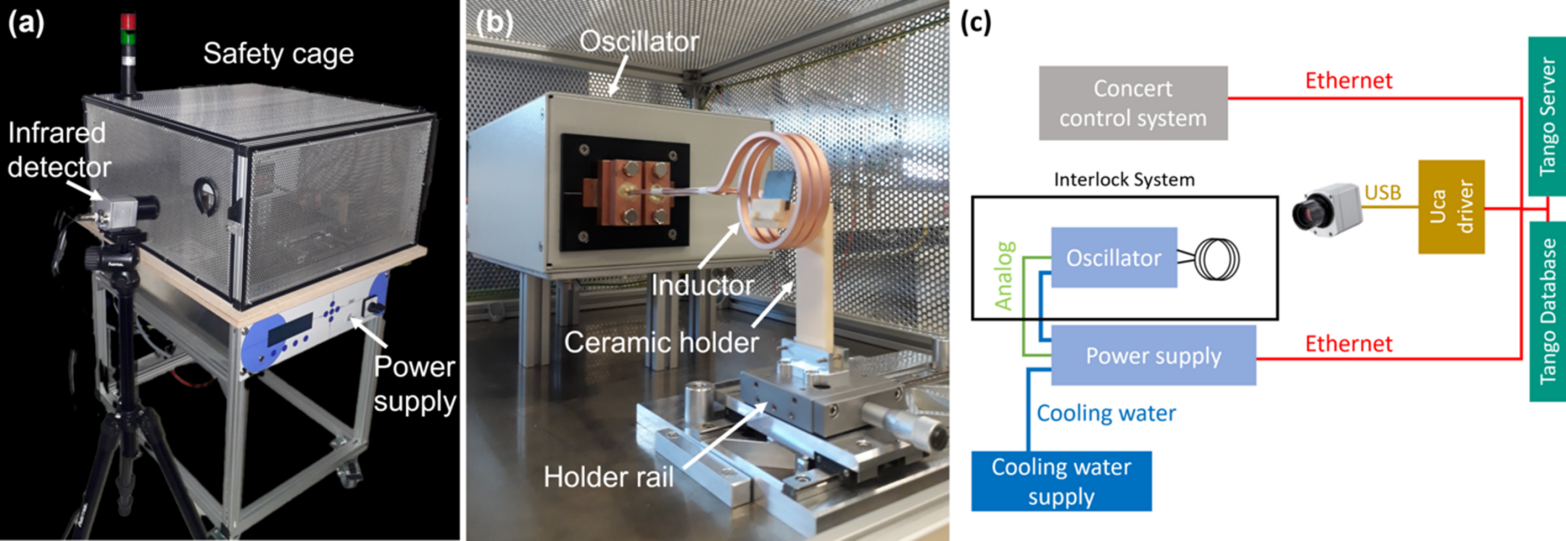
(*a*) Mobile standalone configuration of the induction heating system. (*b*) View of the system inside the safety cage. (*c*) Overall layout including control system integration.

The induction coil is powered by a high-frequency oscillator, enabling rapid and contactless heating of the sample. Electromagnetically induced eddy currents enable volumetric Joule heating of the material, depending on generator frequency and the electrical properties of the material. The applicability of the induction heater depends on the electromagnetic coupling of the sample, in particular its electrical conductivity. Electrically conductive materials and semiconductors can be heated efficiently, whereas highly insulating materials require indirect heating, *e.g.* via thermal coupling to surrounding conductive components. The system was tested for electromagnetic compatibility, including emissions and electromagnetic field measurements, and complies with CE requirements, indicating compatibility with operation in environments with sensitive instrumentation. The system enables undisturbed temperature monitoring by imaging the sample’s own thermal emission using an infrared camera during the heating process. The core components of the induction heating system are shown in Figs. 1 ▸(*a*) and 1(*b*), while the overall layout is illustrated in Fig. 1 ▸(*c*). The compact assembly comprises induction components (power supply, oscillator and inductor), a sample holder, a holder-rail system, and an infrared detector. Only the high-voltage elements are enclosed within a protective cage to ensure safe operation.

The custom-designed inductor coil is fabricated from copper tubing and comprises three concentric turns with outer and inner diameters of 56 mm and 50 mm, respectively, and an axial length of approximately 30 mm. The power supply delivers up to 6 kW (with maximum output of 300 V and 40 A), and water cooling is circulated through the power supply, the oscillator circuit and the copper tube of the inductor coil to ensure thermal stability during continuous operation. The operating frequency can be tuned in the range 10–500 kHz by adjusting the capacitance of the oscillator circuit. For the experiments presented here, a capacitance of 0.33 µF was used, yielding an oscillation frequency of 358 kHz.

The coil is arranged vertically to facilitate synchrotron X-ray diffraction experiments, as shown in Fig. 8(*b*). The maximum heating temperature of approximately 1600°C is primarily limited by the high-temperature capability of the Al_2_O_3_ sample holder, which was selected due to its superior maximum operating temperature compared with alternative ceramics. The inverted L-shaped ceramic holder is designed for flat and laterally extended specimens, such as semiconductor wafers. The sample holder has a fixed tilt angle of 12° with respect to the incident beam, which defines the accessible diffraction geometry for synchrotron experiments. The holder remains unaffected by the inductive heating and provides thermal insulation. A rail system enables translational alignment of the sample with respect to the center of the coil. In the future, motorized stages can be integrated, provided that sufficient distance is maintained from the induction coil to avoid electromagnetic interference.

A near-IR camera (Optris PI 1M, spectral range 850–1100 nm) allows contactless temperature monitoring of the sample surface during operation and enables recording of the temperature distribution across the wafer in a time-resolved manner. The measurable temperature range is 450–1800°C, limited by the spectral range and also depending on the emissivity of the material, *i.e.* for Si (ɛ = 0.67), the effective lower limit is approximately 545°C. The IR camera features an array of 382 × 288 pixels, with the effective spatial resolution at the sample adjustable by varying the working distance.

For standalone operation, personnel safety requires the high-voltage elements to be enclosed within an interlocked protective cage [see Fig. 1 ▸(*a*)]. For experiments within external safety systems providing a suitable interlock signal, as is the case for typical synchrotron beamlines, the power supply interlock system can be connected to the beamline safety interlock system. Therefore, housing can be removed, providing maximum flexibility for *in situ* operation. For additional safety, the system is equipped with an emergency stop button, which allows immediate manual shutdown of the heater in case of any abnormal condition.

### 2.1. Control system and modes of operation

The heating system is operated via the in-house-developed Python-based control system *Concert* (Vogelgesang *et al.*, 2016 ▸; Concert, 2025 ▸), which provides centralized access to all hardware components and experimental procedures within a unified environment [see Fig. 1 ▸(*c*)]. The underlying communication layer is based on the *Tango* framework (Tango, 2025 ▸), enabling the integration of the heating system into standard beamline control environments. The IR camera and X-ray detector are interfaced via the *libuca* library (Libuca, 2025 ▸) for control and image acquisition.

The system supports two modes of operation: in *programmable mode*, heating parameters (power, current or voltage) can be defined as a function of time, enabling automated and reproducible thermal cycling that can be coordinated with detectors or external triggers; in *interactive mode*, the operator can directly adjust heating parameters in real time, guided by live feedback from the IR camera or X-ray detector. A future implementation will include a *closed-loop mode*, in which the heater output is automatically adjusted based on real-time temperature data to reach or maintain a specified target temperature within a defined region on the sample surface.

### 2.2. Reproducibility of heating conditions

To evaluate the reproducibility of the heating conditions, we heated a silicon wafer sample similar to that described in Section 4.1, *i.e.* approximately 20 mm × 20 mm in lateral size, cleaved from a standard 750 µm-thick wafer. The heating system was operated in programmable mode, applying the same predefined voltage profile *V*(*t*) for each cycle. To assess repeatability under realistic experimental workflows, the wafer was dismounted and remounted between subsequent cycles after cooling down to room temperature. During heating, the time evolution of the 2D temperature field was recorded by the IR camera (1 s exposure time) using a fixed emissivity setting of ɛ = 0.67, valid for silicon in the near-IR band (Satō, 1967 ▸).

**Figure 2:**
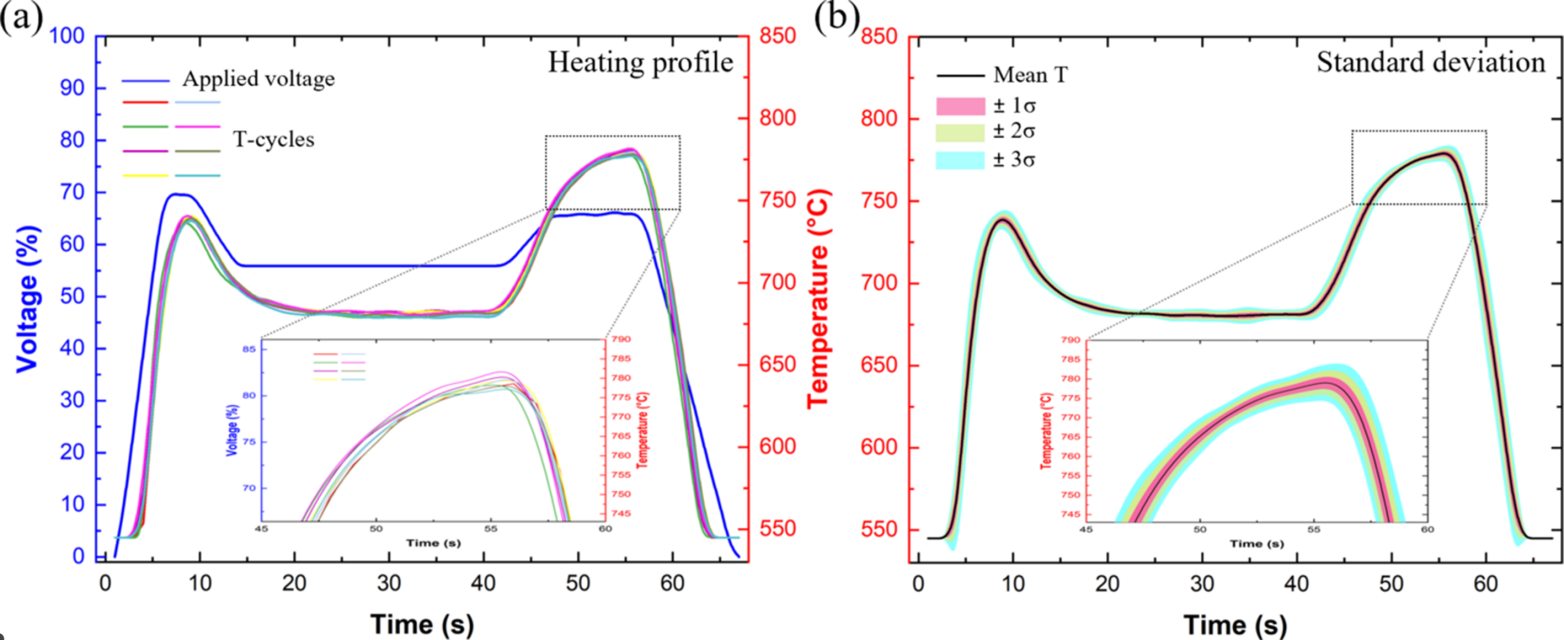
(*a*) Applied generator voltage *V*(*t*) (blue) and the corresponding temperature response for eight consecutive heating cycles. (*b*) Mean temperature (black) with shaded bands indicating ±1σ, ±2σ and ±3σ. The apparent base temperature reflects the lower measurement limit of the IR camera for Si (ɛ = 0.67).

Fig. 2 ▸(*a*) shows the applied voltage profile *V*(*t*) together with the measured temperature responses for eight consecutive heating cycles. For each cycle, the average temperature within a central wafer region was extracted as a function of time. For quantification, the standard deviation σ(*t*) across the eight cycles was computed at each time instant. The statistical evaluation is summarized in Fig. 2 ▸(*b*), where the mean temperature is accompanied by shaded envelopes representing the ±1σ, ±2σ and ±3σ intervals. The time-averaged standard deviation is ±2.7°C, with noticeable deviations occurring mainly during the ramp-up and ramp-down phases. The observed variations reflect both temperature repeatability and temporal reproducibility of the thermal response under repeated mounting conditions.

## 3. Numerical simulation

**Figure 3:**
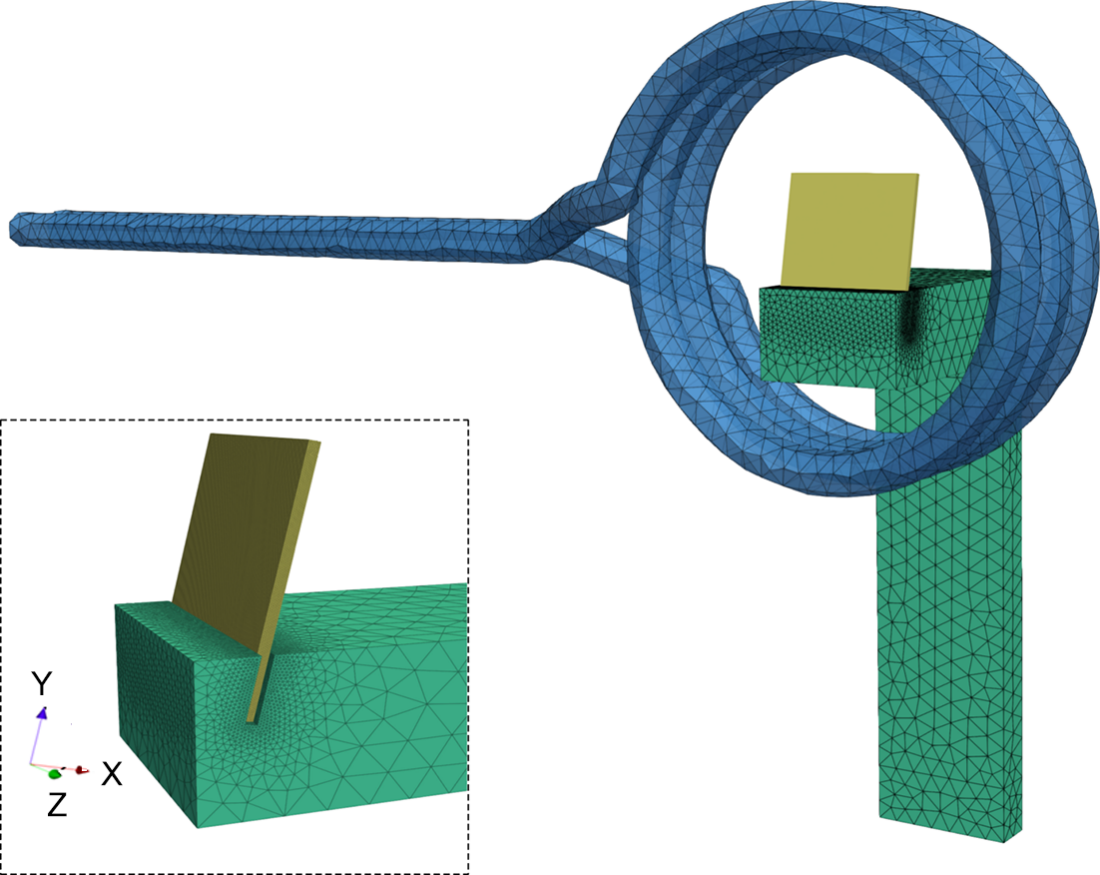
Overall 3D simulation geometry comprising the induction coil, sample holder and sample. The dotted square illustrates the mesh geometry of the sample holder with a silicon wafer of dimensions 18.50 mm × 19.76 mm, thickness 0.75 mm, corresponding to the sample investigated in Section 4.

The geometric scope of the simulation includes the induction coil, the sample holder and a silicon wafer (corresponding to the sample investigated in Section 4), represented in three dimensions as shown in Fig. 3 ▸. The physical scope covers (i) the time-harmonic electromagnetic field to resolve heat generation, (ii) steady-state heat conduction with convective and radiative heat losses, and (iii) thermo-elastic stress analysis with temperature-dependent material properties. The simulations were carried out using the open-source finite element software *Elmer FEM* (Råback *et al.*, 2024 ▸). The source code used for the 2D and 3D models presented in this work will be published as open source (Nemocrys, 2025 ▸). Although the experimental heating protocols are time-dependent, the simulations focused on steady-state conditions corresponding to the maximum temperatures reached during the experiments.

### 3.1. Governing equations

For the present 3D induction heating setup, we employ a time-harmonic formulation – the electromagnetic fields are assumed to vary sinusoidally with angular frequency ω. Expressing the fields in phasor form transforms the problem into a steady-state formulation in the frequency domain. Introducing the magnetic vector potential 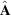 and the electric scalar potential 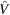, the governing equations are (Lupi, 2017 ▸) 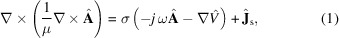

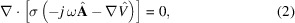
where μ is the magnetic permeability, σ is the electrical conductivity and 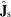 is the source current density.

The induced current density is given by
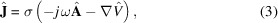
which follows directly from Ohm’s law in phasor form, 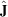 = 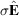, with the electric field expressed as 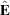 = 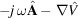. These induced currents, commonly referred to as *eddy currents*, are responsible for resistive heat generation in the conducting regions. The corresponding time-averaged volumetric Joule heat generation, which serves as the source term in the thermal problem, is expressed as
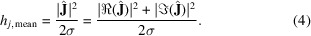
The steady-state heat transfer equation for heat conduction is described by 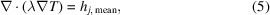
where λ is the thermal conductivity and *T* is the temperature. At the boundaries between the solid and ambient air, the heat balance is modeled considering radiation to the ambient and natural convection cooling, setting the following heat flux boundary condition,
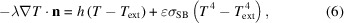
where **n** is the outward unit normal vector, *h* is the heat transfer coefficient, ɛ is the surface emissivity, σ_SB_ is the Stefan–Boltzmann constant, and *T*_ext_ is the ambient temperature.

Thermo-elastic stress analysis under a non-uniform temperature field is described by the generalized Hooke’s law in Voigt notation (Lambropoulos & Delametter, 1988 ▸), 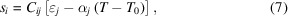
where *s*_*i*_ ≡ 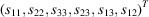 are the stress components, *C*_*ij*_ are the stiffness matrix components, ɛ_*j*_ the strain components, α_*j*_ the thermal expansion coefficients, *T*_0_ is the reference (stress-free) temperature fixed at room temperature, and *T* is the actual temperature. The strain components are obtained from the displacement field *u*_*i*_ via the linearized strain–displacement relation ɛ_*kl*_ = 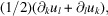 and the displacement field itself follows from the equilibrium equation ∇·*s* = 0 in the absence of body forces.

### 3.2. Model definition

#### 3.2.1. 3D multiphysics solvers

The employed 3D model combines the electromagnetic, thermal and elasticity equations described in the previous section into a single multiphysics simulation, solved with the open-source finite element software *Elmer* (Råback *et al.*, 2024 ▸). The workflow involves three main solvers: the *MagnetoDynamics* module with the *WhitneyAVHarmonicSolver* subroutine for the time-harmonic electromagnetic field; the *HeatSolver* for steady-state heat conduction; and the *Elastic­Solver* for thermo-elastic stress analysis. The sequence is arranged such that the electromagnetic solver first computes the magnetic vector potential 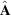 and the electric scalar potential 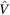 by solving equations (1) and (2). From these, the induced current density 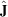 is obtained, and the corresponding Joule heating term *h*_*j*,mean_ is evaluated using equation (4). This heat source is then applied in the thermal solver, which solves equation (5) to determine the steady-state temperature distribution. The resulting temperature field is then passed to the elasticity solver, which performs the thermo-elastic stress calculation according to equation (7).

#### 3.2.2. Geometry and boundary conditions

The geometry of the simulation setup is shown in Fig. 3 ▸. The 3D mesh consists of approximately 1.2 × 10^6^ tetrahedra, where one-third of them are located at the silicon wafer, generated with *Gmsh* (Nemocrys, 2023 ▸; see also https://gmsh.info/). The wafer mesh size is chosen at six elements across its thickness (0.75 mm), which is one order of magnitude smaller than the skin depth of silicon (∼10 mm at 1000°C). The coil region is meshed coarsely, as its electromagnetic behavior is represented by an impedance boundary condition (IBC), similarly to that of Tsiapkinis *et al.* (2024 ▸), and the sample holder is refined only at the interface with the wafer.

The IBC approach assumes that the magnetic field is concentrated within a thin surface layer of thickness given by the skin depth δ = 1/(πμ_0_σ*f*)^1/2^, where μ_0_ is the magnetic constant, σ is the electrical conductivity and *f* is the frequency. The skin depth needs to be much smaller than the characteristic geometric dimensions of the conductor. In this formulation, the tangential electric field at the boundary is related to the magnetic flux density by 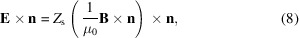
where **n** is the outward normal vector and *Z*_s_ = 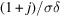 is the surface impedance. This formulation effectively imposes the correct field attenuation in the conductor, while reducing the computational cost by avoiding the need to resolve the skin depth in the mesh. Note that the IBC approach could be also applied on the wafer surfaces if the skin layer becomes too thin to be resolved with mesh elements.

At the outside boundaries of the simulation volume, a vector potential of 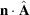 = 0 is set, and 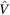 = 0 at the power supplies is prescribed. For the heat transfer, radiative heat losses to the ambient and natural convection cooling were imposed, according to equation (6). The ambient temperature was set to *T*_ext_ = 25°C and the heat transfer coefficient for the wafer and the sample holder to *h* = 10 W m^−2^ K^−1^, representative of quiescent air, which typically lies in the range *h* = 5–25 W m^−2^ K^−1^ (VDI-Gesellschaft Verfahrenstechnik und Chemieingenieurwesen, 2006 ▸). The operation parameters are set to a frequency of 358 kHz, according to experiment, and a current amplitude of 580 A in order to reach the desirable maximum temperature at the silicon wafer, as determined by the IR camera in Section 4.

The material properties employed in the simulations are summarized in Table 1 ▸. Temperature-dependent values are considered for the thermal and electrical conductivities; for silicon, however, the electrical conductivity for the target wafer temperature is held constant at a value obtained from σ(*T*) = 100 × 10^4.247−(2924/*T*)^ (Fulkerson *et al.*, 1968 ▸) with *T* in Kelvin and σ in S m^−1^, as very low conductivity values at low temperatures lead to numerical instability in the electromagnetic solver. The emissivity is set to ɛ = 0.67, matching the value used for the IR camera measurements.

The elastic properties of the silicon wafer are obtained using the temperature-dependent stiffness tensor (Miyazaki *et al.*, 1992 ▸) for cubic crystals, expressed in Voigt notation as *C*_*ij*_ in equation (7), given the cubic symmetry *C*_11_ = *C*_22_ = *C*_33_, *C*_12_ = *C*_13_ = *C*_23_, *C*_44_ = *C*_55_ = *C*_66_. As we can see from Fig. 3 ▸, the *y*-axis is adapted to the 12° inclination of the wafer in order to align the simulation with the crystallographic direction [110]. However, a further θ = 45° rotation about the *x*-axis is required to correctly set the stiffness matrix *C*_*ij*_ (see Appendix *A*[App appa]), so the Cartesian *X*,*Y*,*Z* correspond to the crystallographic axes [001], [110], [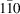]. Hence, the temperature-dependent stiffness matrix coefficients after the rotation are (in GPa) 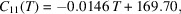

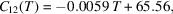

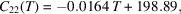

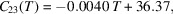

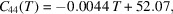

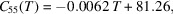
with *C*_12_ = *C*_13_, *C*_22_ = *C*_33_, *C*_55_ = *C*_66_ and *T* in Kelvin.

The applied temperature-dependent thermal expansion coefficient for silicon (Miyazaki *et al.*, 1992 ▸) is given by
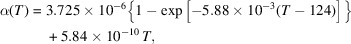
with *T* in Kelvin. For the solution of thermo-elastic stress equations in the wafer, we assume that the wafer’s surface can move freely and that clamping by the sample holder can be neglected.

#### 3.2.3. 2D–3D verification

**Figure 4:**
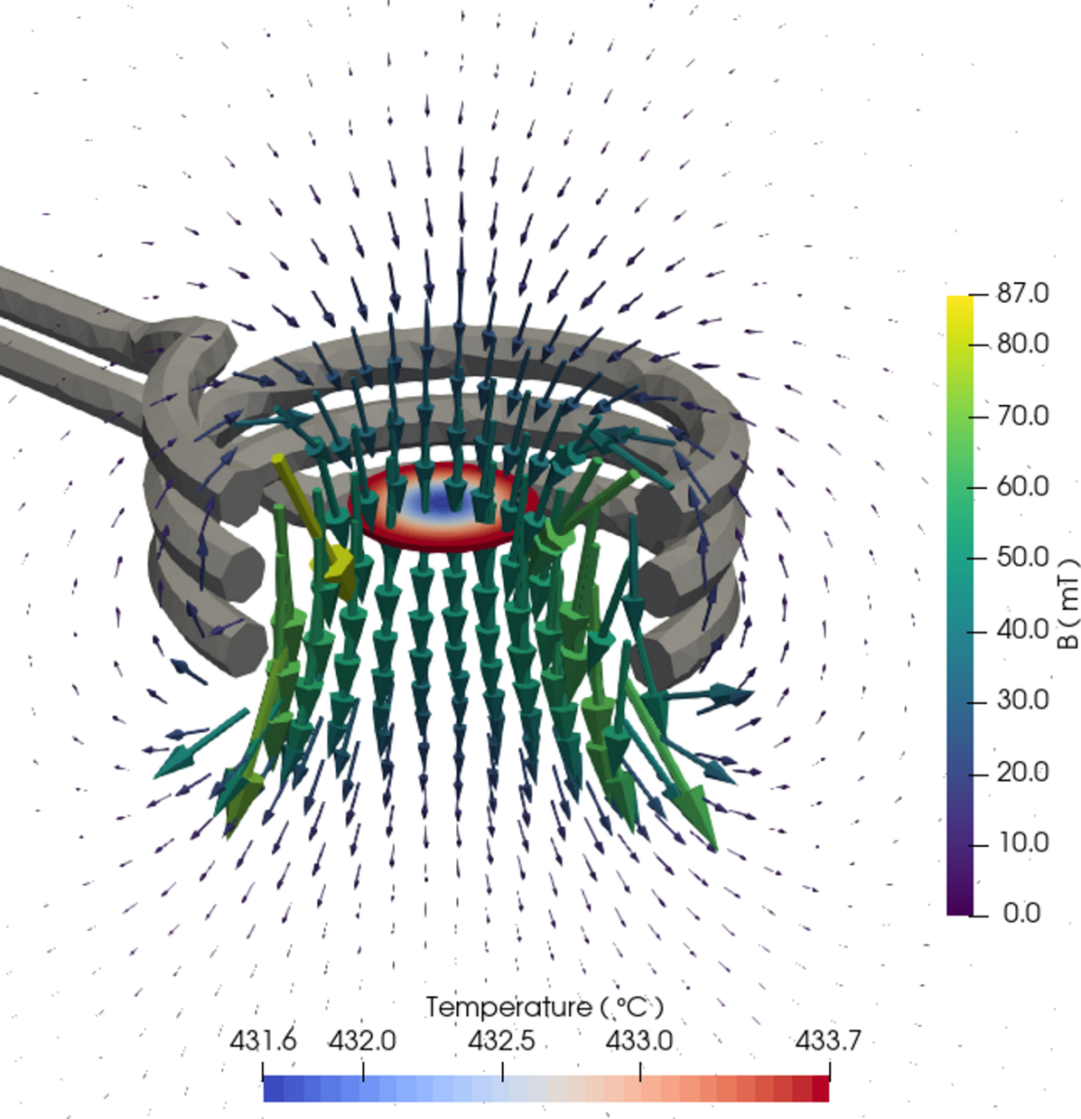
2D–3D verification cases: 3D simulation results. The temperature field of the circular wafer is visualized, while the arrows indicate the direction and magnitude of the magnetic field.

To assess the accuracy of the electromagnetic (EM) and thermal solvers, a comparison was performed between the full 3D model and a simplified 2D axisymmetric model. The 2D verification model consists of a circular silicon wafer, which represents a geometry that can be modeled under axisymmetric assumptions (see Fig. 4 ▸). As the 2D configuration also omits the sample holder, the verification focuses solely on the EM and thermal response of the plate (wafer). The operating point was set to a coil current amplitude of *I* = 800 A at *f* = 358 kHz, with the silicon electrical conductivity fixed at σ = 390 S m^−1^ according to the relation in Section 3.2.2. Under these conditions, the EM skin depth in silicon is approximately δ = 42.5 mm, about 50 times larger than the wafer thickness of 0.75 mm.

Both models use identical material properties and boundary conditions, including impedance boundary conditions for the coil, heat radiation to ambient *T*_ext_ = 25°C, while natural convection losses are omitted for this simplified comparison. The 3D model employs the *WhitneyAVHarmonicSolver* for the time-harmonic EM field, followed by the steady-state *HeatSolver*. The 2D axisymmetric model uses axisymmetric formulations, as described for *MagnetoDynamics2DHarmonic*. The comparison focuses on temperature extrema on the silicon wafer and the induced Joule power.

The results are summarized in Table 2 ▸. The 2D axisymmetric model yields a plate temperature range of 431.8–433.8°C, while the 3D model gives 431.6–433.7°C. The induced power is 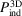 = 6.7 W and 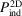 = 6.8 W. All temperature metrics agree within 0.05%, and the total induced power agrees within 1.5%.

### 3.3. Validation of the temperature field

**Figure 5:**
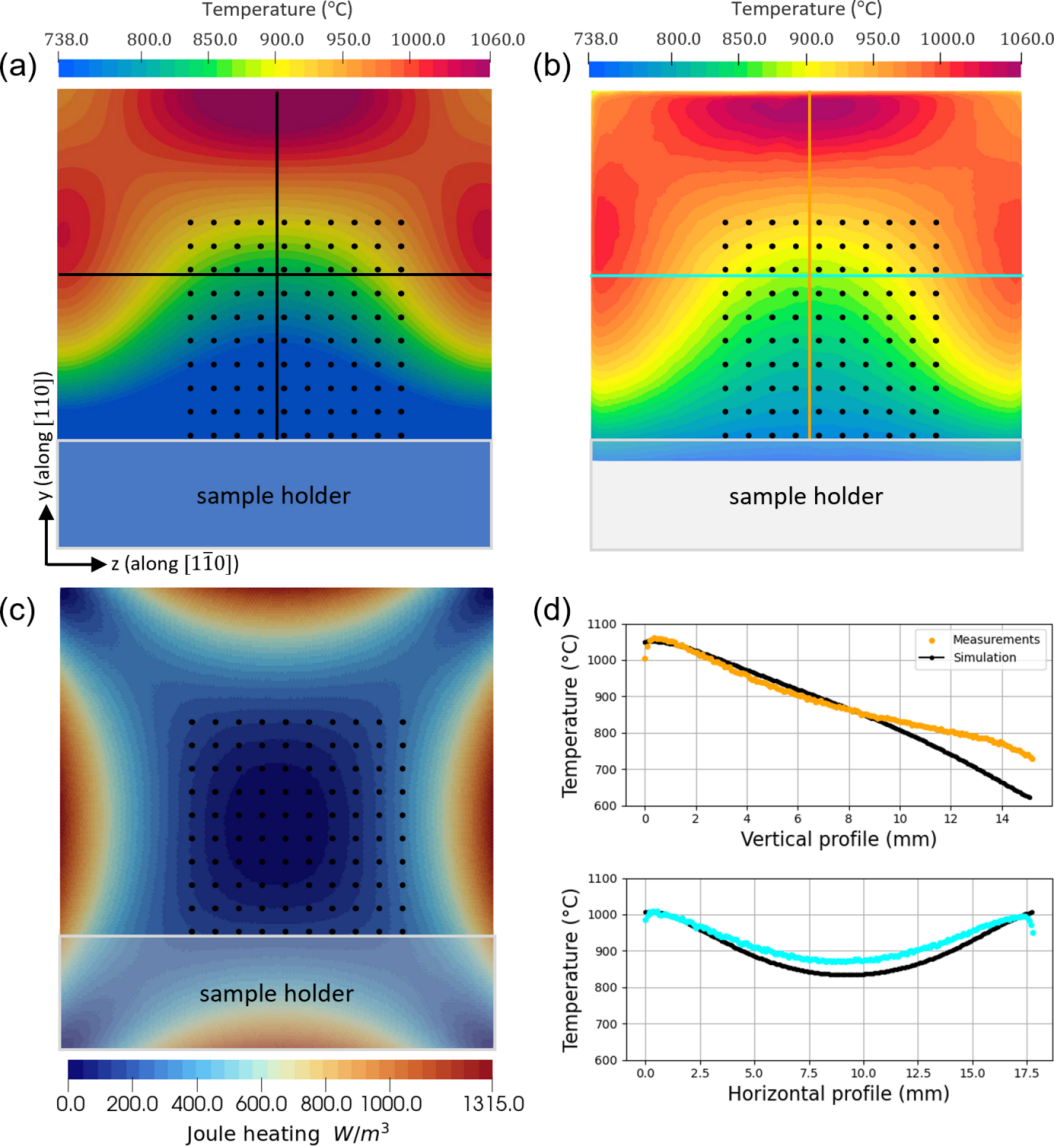
(*a*) Simulated and (*b*) experimental temperature field of the wafer, using the same colorbar scaling. The orange and blue lines are the reference vertical and horizontal lines. The simulated temperature field represents the entire wafer, including the region embedded in the sample holder, whereas the measurement captures only the area outside the holder. (*c*) Induced Joule heating. (*d*) Quantitative comparison of simulation and measurements across the vertical (orange) and horizontal (blue) lines. The positions of the Berkovich indentations are indicated by black dots.

The results of the steady-state simulation according to the experimental setup, corresponding to the maximum temperature of ∼1060°C for the silicon wafer, are shown in Figs. 5 ▸(*a*) and 5(*c*). The temperature distribution exhibits a strong vertical thermal gradient, with the highest temperatures (up to 1060°C) observed in the upper middle region and decreasing towards the bottom, reaching a minimum of 530°C. Along the central vertical profile of Fig. 5 ▸(*a*), this corresponds to an average axial gradient of ∂*T*/∂*y* ≃ 30°C mm^−1^ over the 0–15 mm range. The upper hotter region represents a region of intense induction heating with heat losses due to heat radiation and natural convection, while the bottom cooler part transfers heat via conduction to the ceramic holder. The temperature field distribution is in agreement with the measurements [Fig. 5 ▸(*b*)], as very similar ‘sinusoidal’ shape isotherms at the wafer’s middle and also upper hot region are observed; see also the temperature profiles in Fig. 5 ▸(*d*).

The agreement between simulation and experiment indicates that the steady-state assumption is adequate. The calculated temperature distribution is strictly valid only for the specific wafer material and geometry. However, it can be expected that the main features remain similar if the skin-depth-to-wafer-thickness ratio is similar.

### 3.4. Thermal stress

**Figure 6:**
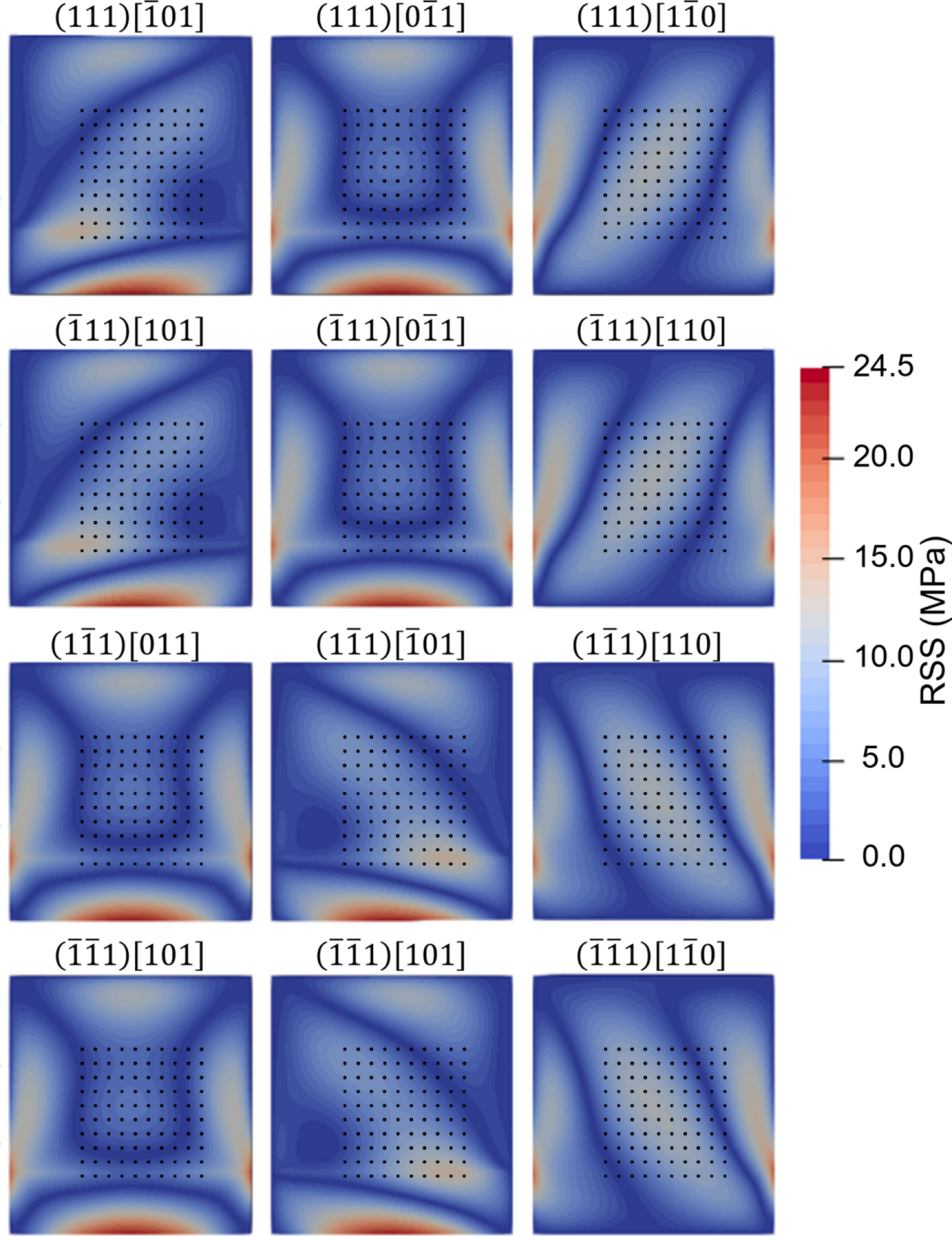
Maps of the absolute value of the RSS for the 12 slip systems of the silicon wafer sample. The distributions are shown over the full sample area in sample coordinate system (*x*, *z* in mm, see Fig. 3 ▸). The positions of the Berkovich indentations are indicated by black dots.

Resolved shear stresses are evaluated here as a driving force indicator for dislocation glide, providing a relative measure for comparing different {111}〈110〉 glide systems for a given thermal state. The simulated steady-state temperature distribution was converted into elastic stress due to thermal expansion via equation (7). Fig. 6 ▸ shows the resulting spatial maps of absolute RSS for the 12 glide systems in silicon as obtained by the relationships given in Appendix *B*[App appb]. Each subplot corresponds to one slip system, with the slip direction and planes indicated above on each plot. As expected, some of the RSS maps exhibit equivalent stress distributions due to the crystallographic symmetry of the diamond-cubic lattice.

More specifically, an almost homogeneous temperature field is observed over the silicon wafer depth, *i.e.* along the *x*-axis. Hence, out-of-plane stress components with *x*-index are negligible (|σ_*ix*_| ≃ |σ_*xj*_| 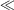 |σ_(*i*≠*x*,*j*≠*x*)_|), simplifying the stated expressions in Appendix *B*[App appb] to a set with six equations. Among these six, two appear as sign-reversed pairs, since for any slip system (**n**, **b**) the crystallographically equivalent system with the opposite Burgers vector (**n**, −**b**) satisfies τ_**n**,−**b**_(*y*, *z*) = −τ_**n**,**b**_(*y*, *z*). As activation of the dislocation glide is governed by the Schmid criterion, the relevant quantity is the magnitude |τ_**n**,**b**_|, while the sign simply determines the glide direction. Consequently, slip systems related by τ_**n**,−**b**_ = −τ_**n**,**b**_ yield the same spatial distribution when plotted as absolute RSS.

## 4. Application: time-resolved observation of dislocation dynamics

Plastic deformation in semiconductors is governed by dis­location glide (Yonenaga & Sumino, 1978 ▸), where handling-related surface or edge damage may act as sources for thermally activated dislocations during subsequent treatments (Tanner *et al.*, 2011 ▸; Tsoutsouva *et al.*, 2018 ▸). To evaluate the capability of the induction heating system and the numerical model to investigate stress-induced plastic deformation in semiconductor wafers in realistic conditions, we examined a Si(001) wafer containing a defined array of Berkovich indents. During controlled induction heating, we simultaneously monitored the wafer by IR thermography and X-ray white beam topography. This experimental design provides (i) well defined dislocation sources and (ii) direct access to both the local temperature field and the resulting diffraction contrast. The main objectives are to determine the onset temperature of dislocation motion, to identify the active {111}〈110〉 glide systems, and to correlate the observed nucleation and propagation of dislocations with the simulated RSS distributions.

Using the interactive mode of the induction heating system, we applied stepwise voltage set points to identify the temperature range that initiates and sustains dislocation motion. Owing to its compact, mobile design, the system integrates into the beamline infrastructure and enables simultaneous and time-resolved acquisition of XWBT and IR data.

XWBT employs a polychromatic beam to form a Laue pattern with multiple *hkl* reflections. Each diffracted beam produces a 2D topograph, *i.e.* a projection image in which the diffraction contrast encodes lattice distortions such as near those extended defects, integrated along the diffracted-beam direction through the illuminated volume (Raghothamachar *et al.*, 2006 ▸). Recording image sequences with an X-ray digital detector provides time-resolved movies; the frame rate is limited by the exposure time needed to collect sufficient X-ray signal (Danilewsky, 2020 ▸).

### 4.1. Sample preparation

**Figure 7:**
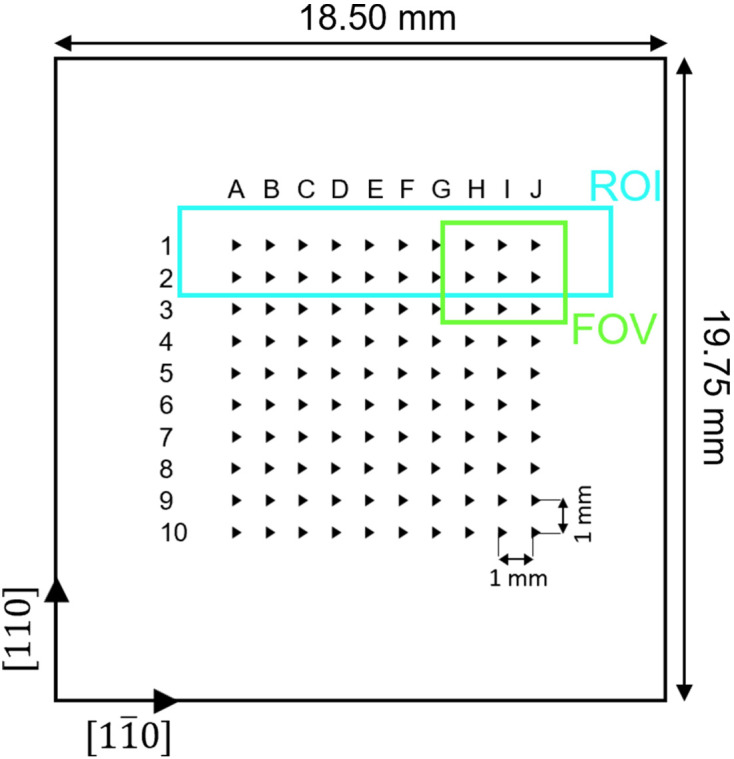
Schematic of the investigated Si wafer with an array of 10 × 10 Berkovich indentations at its center. The indents are referenced A–J (left–right) and 1–10 (top–bottom). The green rectangle indicates the FOV imaged during the *in situ* XWBT measurements; the blue box marks the ROI characterized *ex situ* after heating.

For this study, a silicon (Si) sample measuring approximately 18.5 mm × 19.75 mm was cleaved along the crystallographic directions [110] and 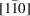 from a commercially available, dislocation-free p-doped Si(001) wafer (diameter 200 mm, thickness 750 µm) grown by the Czochralski method. At the center of the (001) surface, we introduced a 10 × 10 array of Berkovich indentations with a pitch of 1 mm in both directions; see Fig. 7 ▸ and Section 4.3. Each indentation was applied at a maximum load of 400 mN, with one edge of the Berkovich triangle aligned parallel to the [110] direction.

### 4.2. Experimental setup

All *in situ* and *ex situ* XWBT experiments were performed at the topography station of the Imaging Cluster of the KIT Light Source, Karlsruhe Institute of Technology. The station uses the continuous, polychromatic spectrum of a bending-magnet source. Apart from upstream slits for beam shaping, the only optical element in the X-ray path is the beryllium exit window, which minimizes distortion of the beam profile and ensures homogeneous illumination for full-field X-ray diffraction imaging.

**Figure 8:**
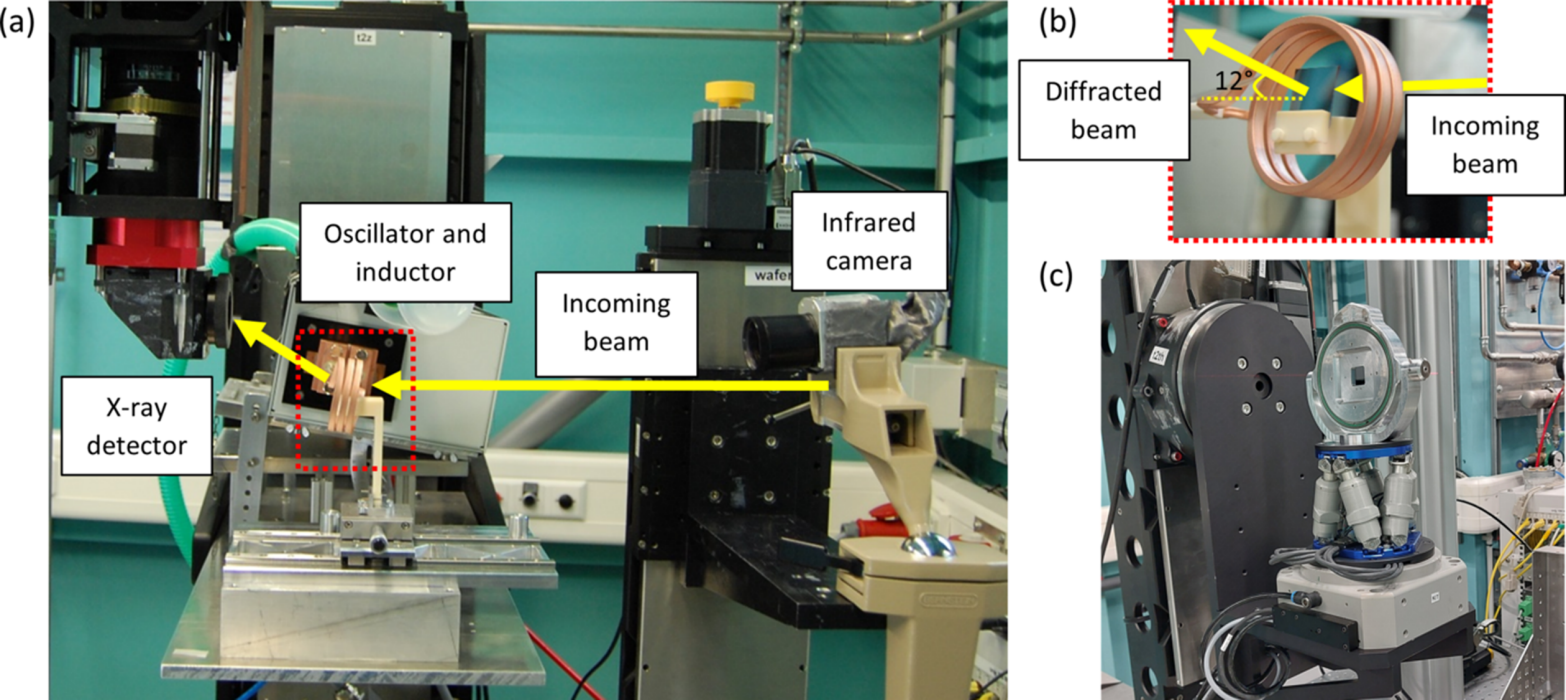
XWBT setups used for the measurements at the KIT Light Source. (*a*) *In situ* sample environment installed on a translation stage. The X-ray detector and the IR camera observe the sample simultaneously. (*b*) The sample positioned in the center of the induction coil. (*c*) *Ex situ* setup enabling flexible sample positioning and orientation.

As shown in Fig. 8 ▸(*a*), the oscillator, the induction coil and the rail-mounted sample holder were installed on a common base plate on top of a stacked pair of motorized translation stages oriented perpendicular to the beam (*X* horizontal, *Z* vertical). Since the X-ray beam and detector field of view are fixed and smaller than the wafer size, the system was mounted on translation stages to position different regions of the sample within the beam. This configuration allows precise positioning of the induction heating system to select a region of interest (ROI) during *in situ* studies and, alternatively, stitching of several regions to image large sample areas (wafer mapping) by translating the stage before or after annealing, hereby enabling an overview of the entire sample after each heating cycle. Integration of the induction heating system into the beamline control infrastructure via the *Tango/Concert* framework (see Section 2.1) enabled unified control of XWBT and IR data, including control of the translation stages and ancillary safety systems (*e.g.* interlock systems and beam shutter). All *in situ* measurements were performed on a common time base, ensuring consistent experimental control and data acquisition.

The indirect X-ray detector system employed a 200 µm-thick LuAG:Ce scintillator, optically coupled to a digital camera through a 3.6× visible-light objective. Two cameras were used: (*a*) a pco.edge 5.5 equipped with an sCMOS sensor (2560 × 2160 pixels, 6.5 µm pixel pitch), resulting in an effective pixel size of 1.8 µm. The camera allowed exposure times of 0.01–1 s, which were suitable for the *in situ* experiments, enabling time-resolved imaging at sub-second cadence; and (*b*) a pco.4000 with a CCD sensor (4008 × 2672 pixels, 9 µm pixel pitch), yielding an effective pixel size of 2.5 µm. It allowed exposure times longer than 1.4 s and was therefore used for the *ex situ* measurements requiring high signal-to-noise ratios, for example, for weakly diffracting reflections.

Time-resolved XWBT was performed in the 220 reflection. The sample was inclined by a fixed angle of 12° relative to the incident beam [see Fig. 8 ▸(*b*)]. Neglecting wafer miscut, this angle equals the Bragg angle in coplanar geometry, which, according to the Bragg condition, corresponded to an X-ray energy of 15.5 keV for diffraction. Using the beamline slits, the beam size was adjusted to approximately 4.3 mm × 3.5 mm, slightly smaller than the FOV of camera (*a*). The sample–detector distance was approximately 110 mm. The IR camera viewed the opposite wafer surface with its optical axis aligned with the surface normal. The sample–camera distance of 335 mm resulted in an effective pixel size of 99.4 µm and a field of view of 38.4 mm × 28.9 mm. For both the X-ray detector and the IR camera, the exposure time was set to 0.25 s. Similar to Section 2.2, an emissivity of ɛ = 0.67 was used for the IR camera (Satō, 1967 ▸).

After the thermal treatments, *ex situ* XWBT measurements were performed. For these, the induction heating system was replaced by the standard tomography setup. The setup consists of, from top to bottom, (i) a manual Eulerian cradle; (ii) a hexapod; (iii) a rotation stage with vertical axis; and (iv) a rotation about the horizontal axis (perpendicular to the beam) [see Fig. 8 ▸(*c*)]. This allowed flexible positioning and orientation, providing access to multiple reflections (220, 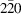, 400, 040, 311, 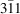, 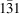, 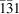) while keeping the sample mounted. The sample–detector distance was approximately 150 mm and the beam size was adjusted to approximately 7 mm × 4.5 mm.

### 4.3. Experimental results

The Si wafer was subjected to two successive time-resolved *in situ* thermal treatments. Here, the interactive mode of the induction heating system was used by adjusting the generator voltage during simultaneous IR and XWBT imaging. During the first thermal treatment, this allowed for direct exploration of the thermal response of the sample, in particular the determination of the temperature *T*_glide_ and the corresponding heater voltage at which the onset of dislocation glide occurred. Based on this, the second treatment was adjusted to bring the sample more quickly to the vicinity of *T*_glide_ and to keep it there for an extended period of time to promote further dislocation activity.

**Figure 9:**
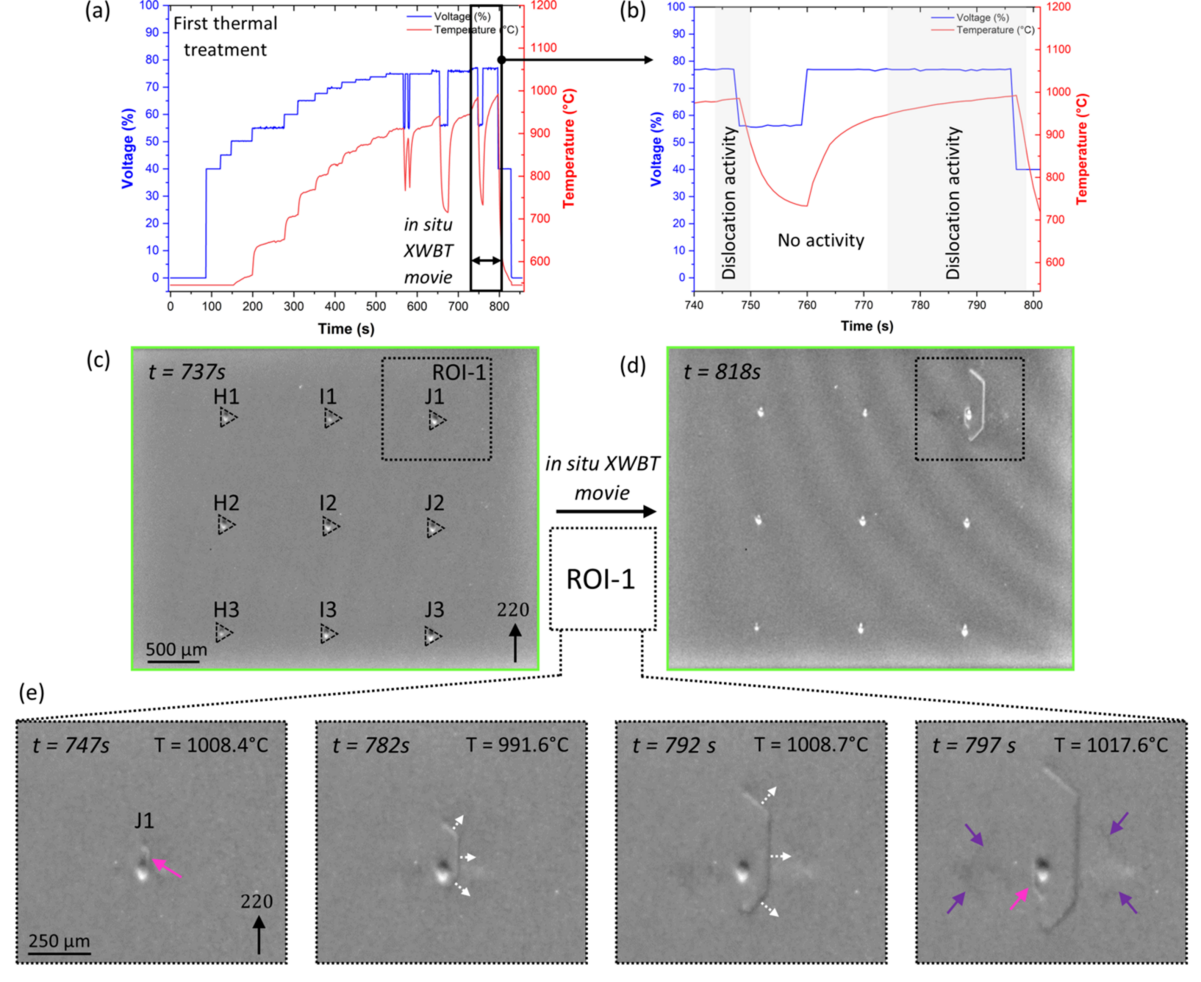
First thermal treatment. (*a*) Applied generator voltage *V*(*t*) (blue) and spatially averaged temperature *T*(*t*) (red) within the XWBT FOV (green rectangle in Fig. 7 ▸). (*b*) Enlarged view of the time interval in which dislocation motion occurred. (*c*, *d*) First and final *in situ* XWBT images of the full FOV recorded at *t* = 737 s and *t* = 818 s. (*e*) Intermediate ROI-1 snapshots around indent J1, illustrating dislocation evolution. Magenta arrows mark newly nucleated dislocations visible in the 220 reflection; purple arrows indicate extinct contrast; white dashed arrows denote glide directions.

During the first *in situ* thermal treatment, two distinct periods of dislocation mobility were observed, separated by a ∼25 s interval, where no detectable motion occurred [see Fig. 9 ▸(*a*), *t* = 737–818 s]. The XWBT movie is available in the supporting information (Video 1). The corresponding XWBT frames for this time interval are shown in Fig. 9 ▸(*b*). The first and final images of the full field of view (FOV) are displayed in the green box in Figs. 9 ▸(*b*) and 9 ▸(*c*), and selected frames featuring dislocation motion are shown above as ROI-1. The time stamps and average IR temperatures are annotated for the FOV and ROI-1, and the values refer to spatial means within the respective regions.

Among the nine monitored indentation sites, only indent J1, which corresponds to the region of highest temperature within the FOV (see Fig. 5 ▸), exhibited significant dislocation activity. In the first activity window (*t* = 744–749 s), a single dislocation half-loop [marked by a magenta arrow in Fig. 9(*e*)] appeared once the local temperature reached *T*_glide_ ≃ 1008°C. After reduction of voltage, the temperature dropped below 920°C, and no further activity was observed during the subsequent interval (*t* = 749–776 s). During the second activity window (*t* = 776–799 s), the existing half-loop expanded further (motion directions indicated by the white dashed arrows), and an additional dislocation nucleated once the local temperature again reached a level similar to that before *T*_glide_ ≃ 1030°C. Additionally, several faint contrasts appeared (purple arrows), indicating the emergence of further dislocations with Burgers vectors fulfilling the extinction condition for the 220 reflection (Danilewsky, 2020 ▸).

**Figure 10:**
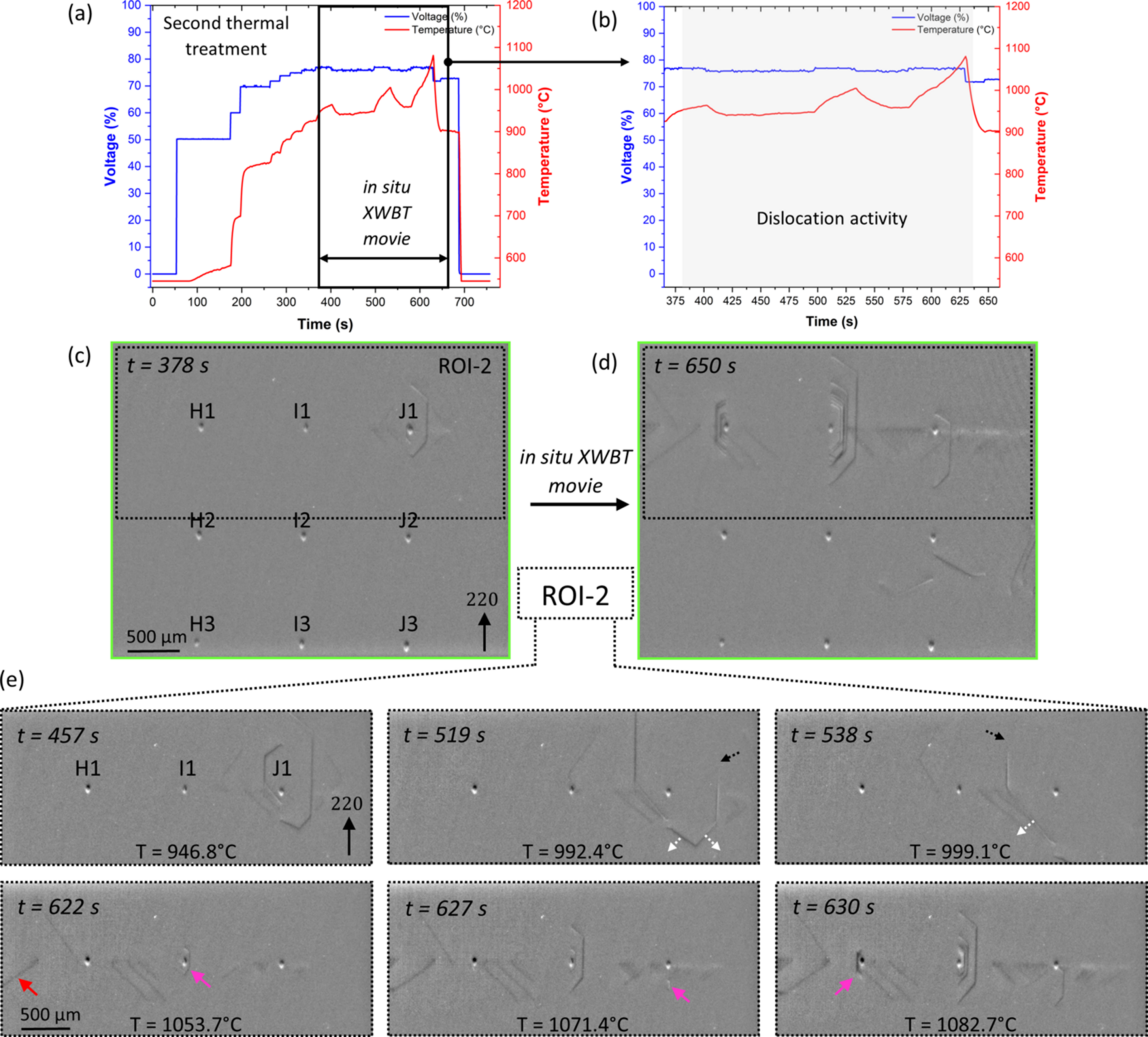
Second thermal treatment. (*a*) Applied generator voltage *V*(*t*) and spatially averaged temperature *T*(*t*) within the XWBT FOV. (*b*) Enlarged view of the time interval with sustained dislocation activity. (*c*, *d*) Initial and final *in situ* XWBT images of dislocation evolution, recorded at *t* = 378 s and *t* = 650 s. (*e*) Intermediate snapshots taken in ROI-2 around indents H1, J1 and I1, illustrating dislocation evolution. Magenta and red arrows mark newly nucleated dislocations visible in the 220 reflection, while white dashed arrows denote the glide directions and black dashed arrows indicate segments that reached the opposite surface and slipped out of the crystal.

Guided by the first thermal treatment, the second thermal treatment adopted a faster stepwise voltage ramp to increase the temperature above 900°C in approximately 350 s and then maintain an elevated temperature to facilitate dislocation motion. A video of the XWBT is available in the supporting information (Video 2). As a result, the second thermal treatment exhibited a single extended period of dislocation activity, indicated in the zoomed panel of Fig. 10 ▸(*a*). Small stepwise adjustments in the applied voltage led to corresponding increases in sample temperature, which ultimately reached a maximum of approximately 1080°C. Fig. 10 ▸(*b*) shows XWBT frames recorded during the dislocation activity interval (*t* = 378–650 s). Fig. 10 ▸(*e*) illustrates the time evolution by a series of XWBT images of a selected ROI labeled ROI-2. The annealing time and average IR temperature are indicated in each image.

During *t* = 457–538 s (*T* = 950–990°C), no new dislocations appeared; instead, dislocations that had already emerged around J1 during the first thermal treatment propagated and extended into the bulk. The dislocation half-loops continued to expand until they reached the opposite sample surface, where surface-parallel segments slipped out (black arrows at *t* = 519 s and *t* = 538 s). The remaining dislocation segments, no longer constrained by closed half-loops, continued to glide, as indicated by the white arrows.

At the temperature peak (*t* = 622–630 s; *T* > 1050°C), the formation of new dislocations was observed near damage sites H1 and I1, and additional segments entered the field of view from the left, indicating activity around indent G1 (red arrow). Notably, at *t* = 627 s, a new dislocation formed slightly below indentation J1, indicating a source within the near-surface volume rather than at the indent apex. As in the case of the first thermal treatment (see Fig. 9 ▸), several faint dislocation contrasts appeared and could be traced across the recorded frames.

**Figure 11:**
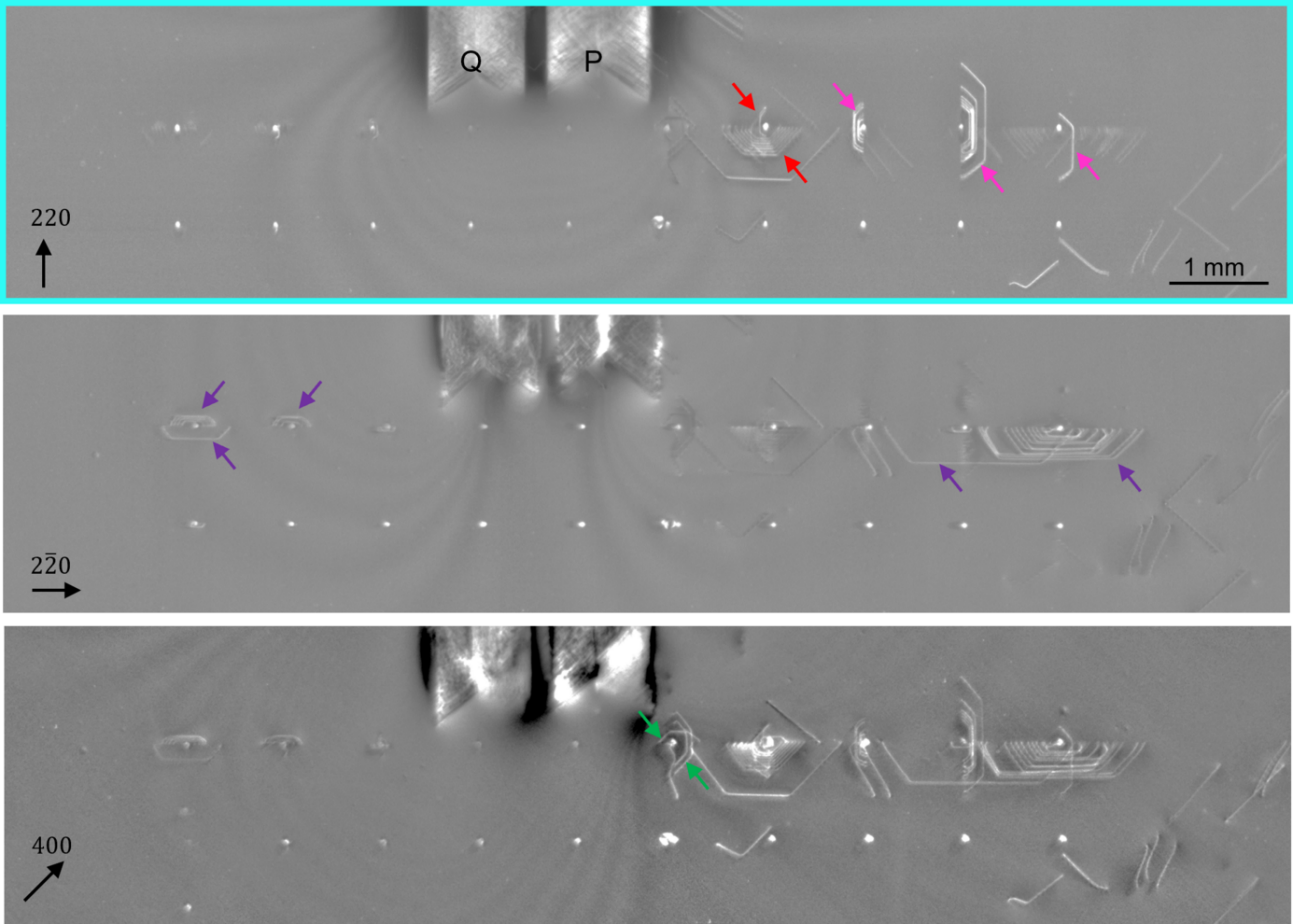
Three of the eight *ex situ* wafer maps (220, 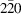 and 400) recorded in the region marked by the blue rectangle in Fig. 7 ▸, showing the final dislocation configuration after both thermal treatments. Variations in dislocation-contrast visibility were used to determine the Burgers vectors using the extinction criterion. Two prominent slip bands, *P* and *Q*, enter the ROI from the wafer edge.

Finally, *ex situ* full wafer mappings revealed that dislocation activity was confined to indentation row 1; only indents D1 and E1 remained inactive. Three of the eight XWBT maps recorded for different *hkl* reflections in the region marked by the blue rectangle in Fig. 7 ▸ are shown in Fig. 11 ▸. By comparing dislocation-contrast visibility across multiple Bragg reflections, the orientations of the associated Burgers vectors were determined using the extinction criterion (Danilewsky, 2020 ▸; Lider, 2021 ▸).

The Burgers vectors and corresponding glide planes of the dislocations, grouped by arrow color, were identified by contrast-extinction analysis from multiple reflections, as summarized in Table 3 ▸. The nucleated slip bands *P* and *Q*, formed at the edge of the wafer, indicate sustained glide on 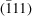 and 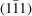 during the thermal treatments.

### 4.4. Correlation to RSS simulations

The *in situ* observations and *ex situ* wafer mappings show that dislocation motion and propagation during the thermal treatments were spatially and crystallographically selective. Thermally induced dislocation activity was confined to indentation row 1, with the exception of indents D1 and E1. Burgers vectors ±[110] and 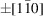 were the most prevalent. In contrast, glide systems associated with directions 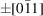 and ±[011] remained inactive throughout the treatments. To relate the observed dislocation distributions to the local driving forces, we computed RSS fields on all {111}〈110〉 glide systems and compared with the experimentally identified Burgers vectors.

**Figure 12:**
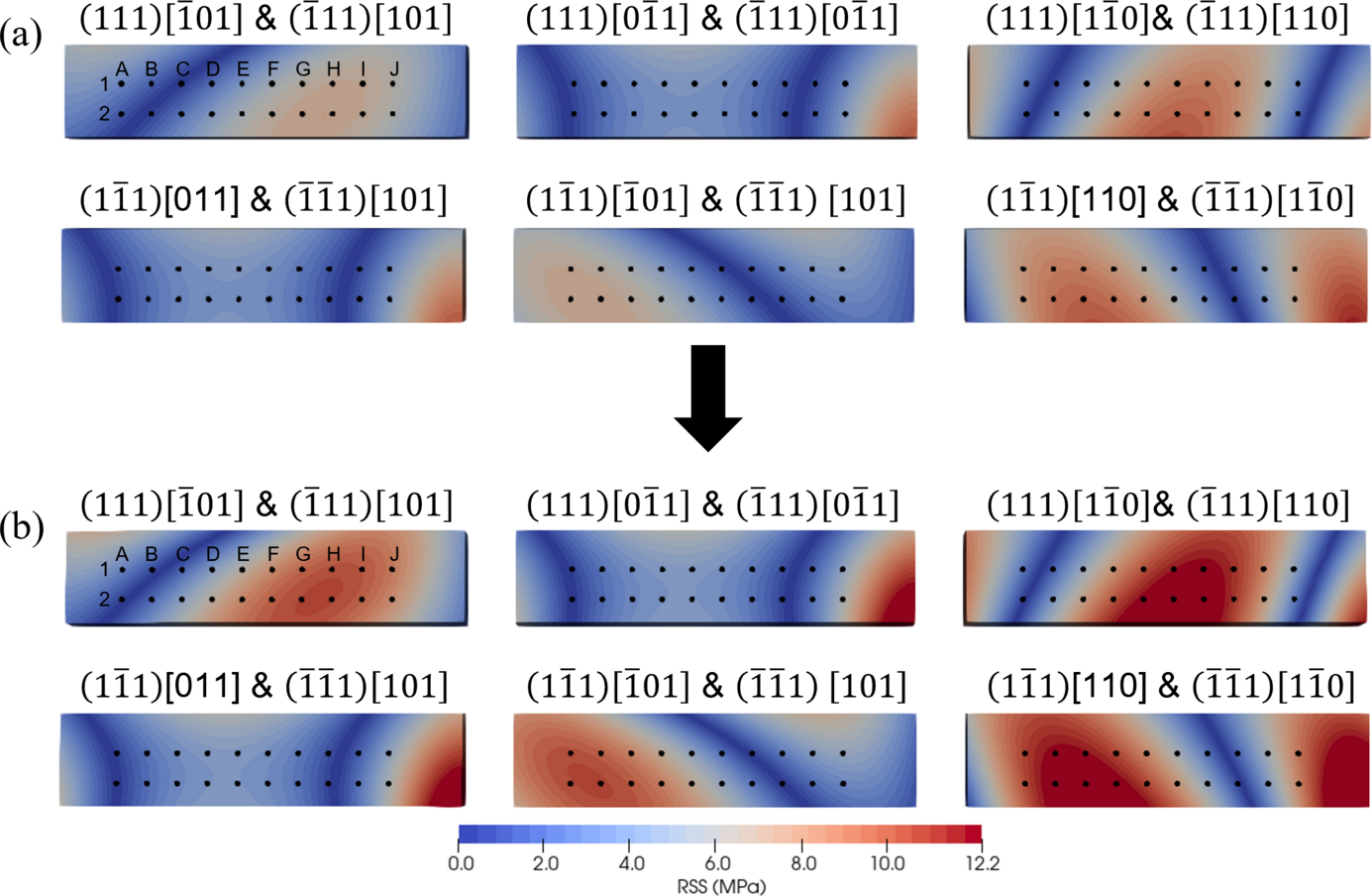
Modulus of the RSS maps for the twelve 111〈110〉 slip systems within the region marked by the blue rectangle in Fig. 7 ▸. Indent position are labeled (A–J, 1–2) to facilitate identification of individual positions. (*a*) First thermal treatment and (*b*) second thermal treatment at the maximum temperatures reached.

Fig. 12 ▸ shows the magnitude of the resulting RSS distributions for both thermal treatments at the maximum temperatures reached within the region marked by the blue rectangle in Fig. 7 ▸. The comparison between the first and second thermal treatments further demonstrates how controlled changes in the thermal conditions influence the characteristics of the dislocation activity. During the first thermal treatment, dis­location motion was observed exclusively at indentation J1 once the local temperature reached about 1000°C. The slip systems 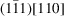 and 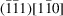, which, according to the calculations shown in Fig. 12 ▸, exhibit the highest RSS magnitudes in the studied region, showed dislocation activity, indicating that only in this region is the combination of sufficient local temperature and RSS reached.

In the second thermal treatment, the temperature in the ROI locally exceeded approximately 1030°C over a larger region of the sample. As a consequence, multiple indentation sites became active, and dislocation motion was observed on several glide systems, such as 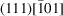 and 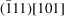. This leads to a broader fulfillment of the activation condition, whereby both sufficient local temperature and RSS exceeded their respective critical thresholds, resulting in enhanced dislocation glide across multiple indentation sites. The observed differences therefore arises from the combined increases in temperature and RSS magnitude, while their spatial distribution remains largely unchanged. This behavior is consistent with the thermally activated nature of dislocation motion, where mobility increases significantly with temperature (Neves Dias *et al.*, 2022 ▸). In the presented experiments, dislocation activity around the indentations first appearing at local temperatures of approximately 1000–1050°C together with RSS values of 8–10 MPa on the preferable {111}〈110〉 glide systems overcome these conditions, while damaged sites below these levels remained inactive through both thermal treatments.

**Figure 13:**
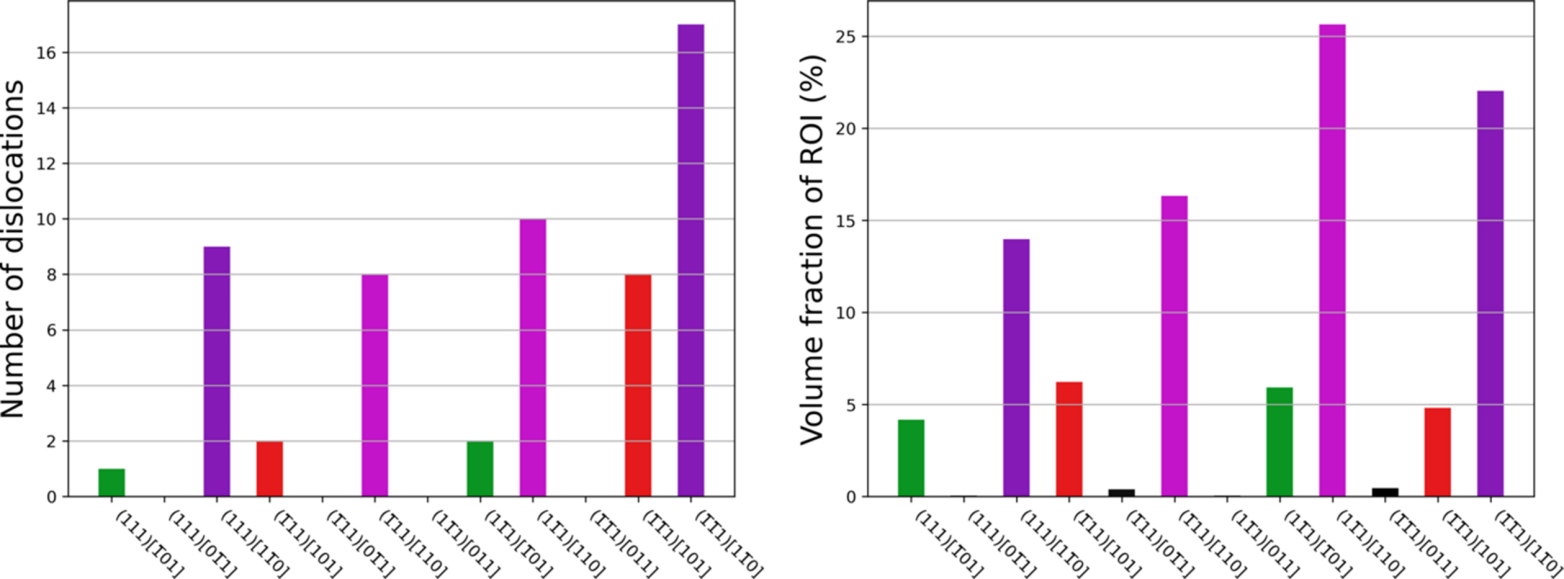
Left: number of dislocations observed experimentally per slip system. Right: histogram of the simulated volume fractions *f*_*s*_ of slip-system activity within the ROI at *T*_max_ = 1080°C. For each mesh point, the slip system with the largest magnitude of resolved shear stress was identified, and the corresponding element volumes were accumulated accordingly. The reported volume fractions represent the normalized contribution of each slip system.

To quantitatively compare the experimentally observed dislocation distributions with the simulations, we compared the number of dislocations observed per {111}〈110〉 slip system with the corresponding simulated volume fractions of slip system activity, where activity is defined by dominance of the RSS magnitude. (Fig. 13 ▸). This evaluation involved three steps: (i) we restricted the domain from the full wafer to an ROI; (ii) at each finite-element mesh point we identified the slip system with the largest RSS magnitude; and (iii) we computed the cell volumes, distributed them to the incident mesh points as representative weights, and accumulated these weighted contributions for each slip system. Specifically, for slip system *s* we define the weighted contribution as 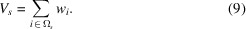
Here, *w*_*i*_ is the representative weight of mesh point *i*, derived from the volumes of its surrounding cells, and Ω_*s*_ denotes the set of mesh points in the ROI that are assigned to slip system *s*. The histogram reports these sums normalized by the total ROI volume as per-system volume fractions, 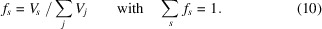
These fractions provide a quantitative measure of the simulated activity of each slip system and can be directly compared with the number of dislocations observed per slip system in the experiment. As shown in Fig. 13 ▸, a clear trend is found: glide systems associated with a larger RSS-weighted volume fraction also show a higher number of experimentally observed dislocations. Although the results were reported for the selected ROI, the same correlations were consistently observed when individual indentation sites were evaluated.

## 5. Discussion

The induction heating system developed in this work is compact and mobile, enabling use in both standalone laboratory environments and at synchrotron beamlines. Eddy-current induction provides rapid, contact-free heating of conductive samples. Based on the integrated IR camera, which monitors the IR emission across the entire sample surface, the setup delivers spatially resolved temperature fields during operation.

A key advantage of the system is its control architecture. Operated through a *Tango*-based interface, the induction heating system is seamlessly integrated into the beamline infrastructure and can be centrally controlled together with other beamline instruments. Simultaneous acquisition of IR and XWBT images, together with the corresponding control parameters, enables direct spatiotemporal correlation between the thermal response and the microstructural evolution. This capability was demonstrated by the time-resolved observation of dislocation dynamics in a silicon wafer at high temperature.

The heater currently supports two complementary modes of operation: programmable heating profiles and interactive manual control. In practice, the interactive mode is valuable during initial experiments for exploration of the critical temperature ranges for dislocation activity, while the programmable mode allows these conditions to be reproduced in subsequent cycles. Since the various material properties that are relevant for observing defect evolution by induction heating depend on the temperature, the materials’ response to the operating parameters is difficult to predict. Hence, manual adjustment during heating is often required to reach and maintain the suitable temperature window. The simultaneous monitoring of temperature and defect distribution provides the feedback needed for this control. A future closed-loop feedback mode based on live temperature data would further strengthen this capability.

The system provides a wide operating temperature range up to about 1600°C with a fast thermal response and high reproducibility. As shown in Section 2.2, repeated heating cycles of a Si sample yield a time-averaged standard deviation of the peak temperature of σ < 3°C, corresponding to only about 0.4% of the total temperature rise of Δ*T* ≃ 700°C. This level of reproducibility indicates sufficient precision for quantitative cycle-to-cycle comparisons of *in situ* studies of defect evolution.

A three-dimensional finite-element model was developed to describe the coupled electromagnetic, thermal and elastic stress fields of the induction heating system. To verify the correctness of the numerical implementation, a reduced two-dimensional model was first compared with the full three-dimensional formulation, and the two approaches yielded consistent results for the key features of the temperature distribution as well as for the induced power. Moreover, the simulated temperature fields agree well with the experimentally measured IR temperature maps (*e.g.* Fig. 5 ▸), despite the steady-state character of the simulations. The qualitative features of the temperature distribution, such as the lateral gradients across the wafer and the relative cooling of the central region, are reproduced, whereas the absolute temperatures and their overall range differ only moderately. The remaining discrepancies between measured and simulated temperature fields are mainly observed near the sample–holder interface, where modeling of the heat transfer is particularly challenging. In this region, the effective heat flux from the sample into the holder depends on several factors, in particular the thermal contact, which is only treated in simplified form in the present model. Following this verification of the general modeling framework, the simulations can be readily adapted to other sample materials, holder geometries and coil designs by adjusting the relevant material parameters and boundary conditions.

The combination of induction heating, IR thermography and numerical modeling provides a basis for the design of the experiment. For the present coil geometry and sample configuration, the resulting temperature field exhibits a slightly cooler central region relative to the periphery. Although a uniform temperature distribution is not desired, control of the thermal gradient is essential for tailoring the stress state and the pattern of dislocation activity. Combined with modeling different coil geometries and operating parameters, the spatial temperature gradients, and thus the resulting stress fields, can be tailored such that the local temperatures and RSS remain below or exceed the critical activation thresholds in selected regions. This enables not only suppression of dislocation activity but also selective activation of dislocations on chosen glide systems.

The application of the heating system for time-resolved *in situ* XWBT demonstrates that the generated thermal stresses are sufficient to activate and move dislocations within the crystal. Onset of dislocation glide was only observed when both the local temperature and the RSS exceeded their respective critical thresholds. Dislocation activity occurred in regions where local temperatures reached approximately 1000–1050°C together with RSS values of 8–10 MPa, on those favored {111}〈110〉 glide systems that meet these conditions, while damaged sites below these levels remained inactive.

Similar time-resolved* in situ* studies based on X-ray diffraction imaging have reported the generation and propagation of dislocations during heating of an intrinsic floating zone single crystal silicon using a Bridgman solidification furnace with two heating zones, highlighting the strong influence of temperature, crystallographic orientation, surface defects and thermomechanical stresses, with glide occurring preferentially along {111} glide planes (Tsoutsouva *et al.*, 2018 ▸). Compared with dislocation propagation temperatures reported in these studies, where dislocations traverse the crystal at temperatures around 1100°C, the lower activation temperature in the present work may result from the higher local resolved shear stresses, or different material properties, such as doping, that effect dislocation mobility.

Dislocation activity was observed exclusively around the first indentation row, which corresponds to higher temperatures than the subsequent rows (see Fig. 5 ▸). The extraction of local temperatures from the simulated temperature field corresponding to the maximum boundary conditions during the second thermal treatment shows that the temperature difference between the first and second indentation rows is approximately 120–145°C. A clear temperature gradient was also recorded along the indentation rows; in the first row, D1 and E1 were located at the cooler end, reaching approximately 1034°C, whereas the average temperature from H1 to J1 was about 1083°C. The lack of observable dislocation activity around D1 and E1 suggests that these indentation sites were close to, but did not fully exceed, the effective activation conditions for dislocation emission during the short peak at the maximum temperature, despite comparatively high RSS on some glide systems, such as the pair of 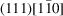 and 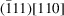, see Fig. 12 ▸. This suggests that maintaining this peak temperature for a slightly longer duration would also activate dislocation emission at these indentations. This is supported by the observed time-dependent activation behavior in Fig. 10 ▸, for example at indentation H1, where dislocations appear only after a few seconds, in contrast to I1, where slightly higher local temperatures lead to more immediate action. This indicates that, in addition to both temperature and RSS magnitude, heating time plays an important role in thermally activated dislocation emission. In contrast, once dislocations are activated, they can propagate at lower temperatures above critical RSS. For example, dislocations activated at J1 were mobilized at temperatures of 950–1000°C.

A complementary perspective is provided by the correlation between the simulated RSS-weighted volume fractions and the experimentally observed slip activity. The analysis in Fig. 13 ▸ shows that glide systems with a larger fraction of the volume experiencing high RSS values also tend to exhibit higher numbers of experimentally observed dislocations. The agreement in the ranking of glide systems indicates that the calculated RSS distribution provides a useful predictor for the relative likelihood of dislocation glide across the different glide systems. However, the concept of comparing RSS-weighted volume fractions with the number of dislocations does not capture all aspects of dislocation behavior. In particular, dislocation interaction, line tension effects and dis­location source mechanisms are not explicitly included in the present model, which may influence the activation and propagation of dislocations. Time-resolved *in situ* imaging data, however, enable a static analysis of dislocation velocities for different types of dislocations, which may allow determination of activation energies for dislocation motion and thus provide experimentally accessible input for more advanced modeling approaches, such as dislocation dynamics simulations.

## 6. Conclusions

Understanding and controlling of dislocation evolution during thermal treatments of semiconductor wafers in industrial processing require *in situ* characterization approaches that combine realistic thermal loads with defect-sensitive X-ray imaging. In this work, we developed a compact, mobile induction heating system designed for time-resolved *in situ* synchrotron X-ray diffraction imaging, which enables contact-free volumetric heating, operable in different modes, up to approximately 1600°C and provides simultaneous access for X-ray and IR imaging. Furthermore, we complemented the instrument with a three-dimensional steady-state finite-element model of the coupled electromagnetic, thermal and thermo-elastic fields and demonstrated its capabilities by studying dislocation generation and motion in a nano-indented Si(001) wafer in a time-resolved manner by synchrotron-based XWBT.

After verification against a two-dimensional reference model and validation against IR thermography, the model reproduces the main features of the spatially varying wafer temperature field. It shows that dislocation activity only occurred when both the local temperature and the RSS exceeded critical thresholds. Furthermore, the model is able to predict the distribution of the RSS on dislocations belonging to the different {111}〈110〉 glide systems. The number of dislocations observed on each glide system was found to correlate with the corresponding RSS-weighted volume fraction from the simulation. This provides a link between dis­location activity and the underlying thermo-mechanical driving forces.

The combination of induction heating, IR thermography, XWBT and numerical modeling provides a quantitative, time-resolved approach to studying defect dynamics under realistic thermal processing conditions. Beyond the demonstration presented here, the modular coil design and simulation-guided optimization will allow the thermal and stress profiles to be adapted for a wide range of materials and wafer geometries. Furthermore, the open and flexible design of the heater makes it attractive also for other kinds of *in situ* studies based on, for example, X-ray diffraction, imaging or spectroscopy.

## Supplementary Material

Supplementary video 1 of the in situ X-ray white beam topography. DOI: 10.1107/S1600577526004728/mad5004sup1.avi

Supplementary video 2 of the in situ X-ray white beam topography. DOI: 10.1107/S1600577526004728/mad5004sup2.avi

## Figures and Tables

**Table 1:** Material properties used in the simulation

Material	Density (kg m^−3^)	Electrical conductivity σ (S m^−1^)	Emissivity ɛ	Thermal conductivity λ (W m^−1^ K^−1^)
Silicon (Fulkerson *et al.*, 1968 ▸; Yamasue *et al.*, 2002 ▸)	2330	9948 @ 1000°C	0.67	1/(2.92 × 10^−5^ + 1.897 × 10^−5^*T* + 1.403 × 10^−8^*T*^2^)
Al_2_O_3_ (Hofmeister, 2014 ▸)	3900	1 × 10^−5^ @ 1000°C	0.8	5.8 @ 1000°C
Copper (VDI-Gesellschaft Verfahrenstechnik und Chemieingenieurwesen, 2006 ▸; Baehr & Stephan, 2010 ▸)	8960	5.8 × 10^7^	0.2	390
Air (VDI-Gesellschaft Verfahrenstechnik und Chemieingenieurwesen, 2006 ▸)	1.19	0	–	25.9 × 10^−3^

**Table 2:** 2D–3D verification cases: comparison between 2D and 3D results for temperature extrema and induced power

Quantity	3D	2D axisymmetric
*T*_min_ on wafer (°C)	431.6	431.8
*T*_max_ on wafer (°C)	433.7	433.8
*P*_ind_ in wafer (W)	6.7	6.8

**Table 3:** Determination of Burgers vectors and corresponding glide planes of color-coded dislocations marked in Fig. 11 ▸, based on contract-extinction analysis across various reflections

Dislocations marked	Visible	Not visible	Burgers vector	Glide planes
Magenta	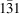 , 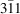 , 220, 311, 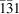 , 040, 400	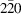	±[110]	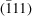 and 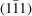
Purple	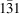 , 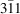 , 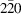 , 311, 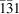 , 040, 400	220	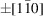	(111) and 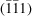
Red	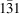 , 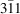 , 220, 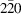 , 311, 400	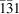 , 040	±[101]	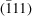 and 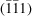
Green	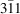 , 220, 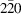 , 311, 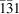 , 040	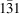 , 400	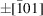	(111) and 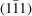

## Appendix A Stiffness matrix rotation

The stiffness matrix for cubic materials, such as silicon, is

where the temperature-dependent stiffness matrix coefficients (in GPa) are (Miyazaki *et al.*, 1992 ▸),

given the cubic symmetry *C*_11_ = *C*_22_ = *C*_33_, *C*_12_ = *C*_13_ = *C*_23_,*C*_44_ =*C*_55_ = *C*_66_. This matrix describes the crystal aligned with the reference coordinate axes.

When the coordinate system is rotated, both stress and strain tensors must transform,

so that the constitutive law  =  remains valid. This requirement leads to the tensor transformation

This is the standard transformation law for a fourth-order tensor under a change of basis (see Nye, 1979 ▸).

Here, the initial rotation matrix **R** is a 3 × 3 matrix describing a rotation by an angle θ about the *x*-axis, with positive θ defined according to the right-hand rule,

In Voigt notation ([ɛ_*xx*_, ɛ_*yy*_, ɛ_*zz*_, γ_*yz*_, γ_*xz*_, γ_*xy*_] with γ_*ij*_ = 2ɛ_*ij*_), the 3 × 3 rotation matrix **R** is lifted to a 6 × 6 transformation matrix **Q**, so that the rotated stiffness matrix is expressed as

The explicit form of **Q** is[Chem scheme1]The notation *R*_*i*α_ denotes the element of the rotation matrix relating the new axis *i* ∈ {*x*, *y*, *z*} to the original axis α ∈ {1,2,3}.

## Appendix B RSS formulas

With **n** denoting the unit normal of the {111} glide planes and **b** the Burgers vectors (expressed in the above coordinate frame), the RSS on the corresponding glide systems is given by

which yields the following explicit expressions for the 12 {111}〈110〉 glide systems (Dobroc˛ka, 1990 ▸; Garagorri *et al.*, 2012 ▸),

All stress components involving *x* vanish if we assume a plane stress state and finally reduce to
